# Predictive nomogram for post-stroke motor dysfunction using fNIRS

**DOI:** 10.3389/fnhum.2025.1526455

**Published:** 2025-12-04

**Authors:** Menghui Liu, Chunxiao Wan, Chunyan Wang, Xinyi Li, Mengmeng Shang

**Affiliations:** 1 Department of Neurological Rehabilitation, Shengli Oilfield Central Hospital, Dongying, Shandong, China; 2 Department of Rehabilitation Medicine, Tianjin Medical University General Hospital, Tianjin, China; 3 Dongying New Journey Geriatric Hospital, Dongying, Shandong, China

**Keywords:** ischemic stroke, upper limb motor dysfunction, functional near-infrared spectroscopy, predictive model, nomo model

## Abstract

**Background:**

There is a lack of objective evaluation tools for assessing upper limb motor dysfunction in ischemic stroke patients (ULMD-IS). This study aimed to develop and validate a diagnostic nomogram for diagnosing the severity of ULMD-IS using functional near-infrared spectroscopy (fNIRS) data.

**Methods:**

This retrospective analysis included 275 ULMD-IS patients at Tianjin Medical University General Hospital. Patients were randomly assigned to a training group (*n* = 193) or a validation group (*n* = 82). The data were preprocessed using HOMER2. In the training group, least absolute shrinkage and selection operator (LASSO) and multivariate logistic regression were employed to identify predictive variables and construct the nomogram. The nomogram’s performance was validated using the area under the receiver operating characteristic curve (AUC), the Hosmer-Lemeshow goodness-of-fit test, calibration curves, and decision curve analysis (DCA).

**Results:**

No significant differences in baseline characteristics, including sex, age, lesion hemisphere, or medical history, were observed between the training and validation groups. LASSO regression analysis identified three independent risk factors: deoxyhemoglobin (HbD) levels in the affected temporal region, total hemoglobin (HbT) levels in the total region, and HbT levels in the unaffected frontopolar region. These factors showed good differentiation ability (training group AUC: 0.766, verification group AUC: 0.861). The model’s goodness-of-fit was confirmed, and it demonstrated a favorable net clinical benefit. Additionally, correlation analysis between these model variables and activities of daily living (ADL) scores revealed no significant relationships (*p* > 0.05 for all variables), indicating that the identified risk factors may not directly influence ADL performance.

**Conclusion:**

This study identified HbD in the affected temporal region, Total HbT levels, and HbT in the unaffected frontopolar region as independent risk factors for diagnosing the severity of ULMD-IS, and a corresponding predictive model was constructed. Given the model’s limited sensitivity, the nomogram should be regarded only as a supplementary reference for objectively assessing post-stroke motor dysfunction; its utility in predicting treatment outcomes and guiding therapeutic choices remains modest and warrants cautious interpretation.

## 1 Introduction

Individuals face an elevated risk of developing cerebrovascular diseases (Chiu et al., 2014), with significantly higher morbidity, mortality, and disability rates observed in those who have suffered an ischemic stroke (He et al., 2014). Motor dysfunction is a prevalent sequela in stroke patients, exerting a substantial negative impact on their ability to perform daily self-care activities and diminishing their overall quality of life. Currently, the clinical evaluation of poststroke motor dysfunction predominantly relies on various assessment scales (Longhi et al., 2016), including the Fugl-Meyer Assessment Scale and the Modified Ashworth Scale (Van Der Velden et al., 2022, Kongwattanakul et al., 2020). Nevertheless, these scale assessment methods have significant subjectivity, and the assessment results for the same patient may vary greatly among different evaluators, affecting the accuracy and reliability of the diagnosis. Moreover, scale assessments generally offer only a general functional score, posing a challenge for in-depth exploration of the specific pathophysiological mechanisms and subtle characteristics of motor dysfunction (Subramanian et al., 2010). Consequently, there is a pressing need for a diagnostic approach that is more objective, precise, and comprehensive in capturing the full extent of poststroke motor dysfunction.

Functional Near-Infrared Spectroscopy (fNIRS) represents an innovative neuroimaging technology that relies primarily on the principle of neurovascular coupling to monitor the activity state of the brain (Teo et al., 2016, Kinder et al., 2022), offering various advantages including non-invasiveness, real-time continuous monitoring capabilities, and relatively high spatial and temporal resolution (Li et al., 2022, Xie et al., 2022, Eastmond et al., 2022). fNIRS measures changes in the concentration of oxygenated hemoglobin (HbO), deoxygenated hemoglobin (HbD), and total hemoglobin (HbT) in the cerebral cortex, reflecting the hemodynamic response to neural activity (Scholkmann et al., 2014). Studies have shown that changes in HbO and HbT are closely related to the intensity and duration of neural activity, while changes in HbD reflect the metabolic demands of local brain regions (Huppert et al., 2009). These hemodynamic indicators not only provide information about regional brain activation but also reveal patterns of functional connectivity between brain regions. Therefore, fNIRS has unique advantages in assessing post-stroke motor dysfunction, providing detailed information about regional brain activation and functional connectivity patterns (Pinti et al., 2020). Importantly, fNIRS is particularly suitable for patients with upper limb motor dysfunction following ischemic stroke (ULMD-IS), who often have limited mobility. Traditional imaging techniques such as CT and MRI can be quite challenging for patients and their families, especially since MRI has a low tolerance for motion artifacts and requires high patient compliance. In contrast, fNIRS is portable, easy to operate, and has a high tolerance for motion artifacts, making it more suitable for imaging in patients with ULMD-IS.

Current fNIRS research on poststroke motor function tends to focus on functional connectivity between regions of interest (ROIs), while neglecting the functional connectivity of the ROIs themselves (Cai et al., 2024, Liu et al., 2015, Almeida et al., 2017). Research has shown that the recovery of motor function after stroke is closely related to the hemodynamic response in the cerebral cortex. For example, increases in HbO and HbT are typically associated with enhanced regional brain activation, while decreases in HbD reflect reduced metabolic demands in local brain regions (Huppert et al., 2009). Additionally, patterns of functional connectivity between brain regions play a crucial role in motor recovery. Studies have shown that the recovery of motor function after stroke is closely related to increased strength and complexity of functional connectivity between brain regions (Grefkes and Fink, 2014). Therefore, monitoring changes in HbO, HbT, and HbD using fNIRS can provide a deeper understanding of the pathophysiological mechanisms of post-stroke motor dysfunction and offer important biomarkers for motor recovery (Pinti et al., 2020).

Our research focused not only on the entire brain but also pays attention to the correlation between functional connectivity within the ROI and the severity of ULMD-IS. Previous studies have shown that the recovery of motor function after stroke is closely related to increased strength and complexity of functional connectivity between brain regions (Grefkes and Fink, 2014). For example, Grefkes (Grefkes et al., 2008) found that the recovery of motor function after stroke was associated with enhanced functional connectivity between the affected and unaffected hemispheres. Additionally, Pinti (Pinti et al., 2020) demonstrated that changes in HbO and HbT monitored by fNIRS could effectively predict motor recovery in stroke patients. Therefore, monitoring changes in HbO, HbT, and HbD using fNIRS can provide a deeper understanding of the pathophysiological mechanisms of post-stroke motor dysfunction and offer important biomarkers for motor recovery.

In this study, we specifically focused on different types of functional connectivity edges to more finely describe the structure of brain networks. Specifically, “Short1 of edges” refers to intrahemispheric connections, that is, the number of edges between nodes within the same region of interest (or subnetwork). “Short2 of edges” refers to intrahemispheric connections, that is, the number of edges between nodes in different regions of interest (or subnetworks). “Long1 of edges” refers to interhemispheric connections, that is, the number of edges between nodes in symmetric regions of interest (or subnetworks) between the left and right hemispheres. “Long2 of edges” refers to interhemispheric connections, that is, the number of edges between nodes in asymmetric regions of interest (or subnetworks) between the left and right hemispheres (Zhu et al., 2017). These classifications help us gain a deeper understanding of the organizational structure of brain networks and how they are related to motor dysfunction after stroke.

The goal of this study was to develop a nomogram based on resting-state brain functional connectivity for diagnosing motor dysfunction after stroke. We comprehensively collected resting-state fNIRS data and relevant clinical information, including age, sex, disease progression, and lesion location, from stroke patients. The innovation of this study lies in the fact that we not only identified key indicators with significant diagnostic value, but also developed a diagnostic model using statistical methods and transformed it into an intuitive and visual nomogram. The clinical value of this visual predictive model (nomogram) lies in its ability to simplify complex diagnostic models into an easily understandable and operable format for use by clinicians in practical settings. We carefully validated and optimized the model, which is expected to significantly improve the accuracy and efficiency of diagnosis, advancing the application and development of neuroimaging technology in clinical practice.

## 2 Materials and methods

### 2.1 Participant recruitment

**FIGURE 1:**
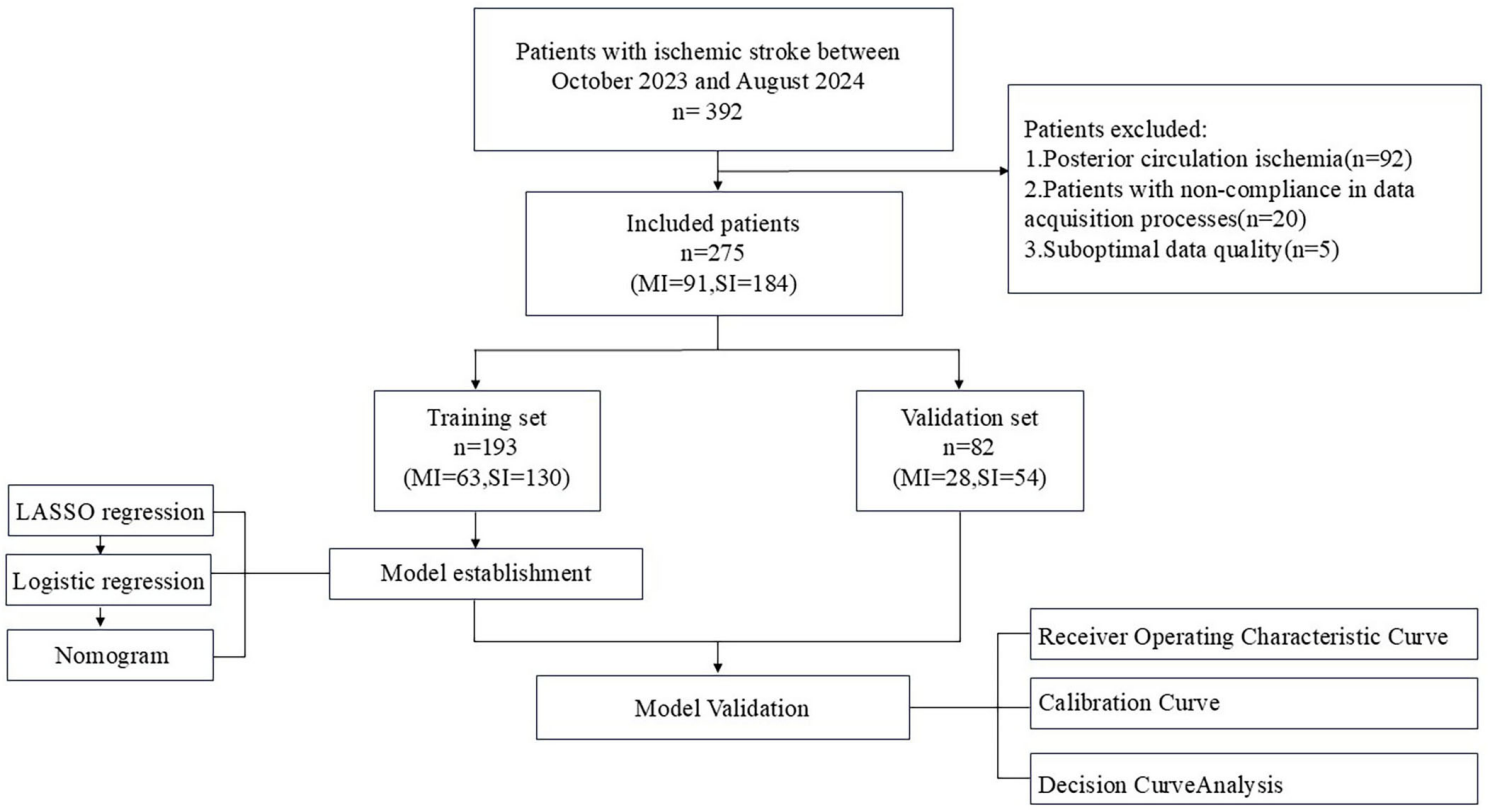
Flow diagram of the study.

This study received approval from the Ethics Committee of Tianjin Medical University General Hospital (Ethics Number: IRB2022-YX-054-01) and adheres to the principles outlined in the Declaration of Helsinki. Given its retrospective research design, the study was granted a waiver for obtaining informed consent from the participants. All patient medical data were anonymized and deidentified prior to analysis. To ensure patient privacy and ethical compliance, the following measures were taken: (1) Data anonymization: All patient identifiers were removed from the dataset, and the data were coded using unique identifiers that could not be traced back to the patients. This process was supervised by a dedicated individual to ensure that no personal information was inadvertently disclosed. (2) Data access control: Access to the anonymized dataset was strictly limited to research team members who had undergone ethics training and signed confidentiality agreements. The data were stored on a secure server with restricted access, and all data transfers were encrypted to prevent data breaches. (3) Ethical oversight: The study was subject to ongoing ethical oversight by the hospital’s ethics committee, which reviewed the study’s progress and ensured that all ethical guidelines were followed throughout the research process. Any potential ethical issues were promptly addressed and resolved. (4) Patient benefits: The study aimed to improve the diagnosis of upper limb motor dysfunction in ischemic stroke patients, which could potentially benefit future patients. The waiver of informed consent was justified on the grounds that the study posed minimal risk to participants and had the potential to significantly contribute to medical knowledge and patient care. The study involved screening data from all patients who were diagnosed with ischemic stroke and admitted to the Rehabilitation Department of Tianjin Medical University General Hospital between October 2023 and August 2024; 392 patients were included. The inclusion criteria for the study were as follows: (1) A confirmed diagnosis of ischemic stroke established through MRI or CT imaging; (2) Provision of informed consent by the patient or their authorized representative; and (3). Age of 18 years or older. The exclusion criteria were as follows: (1) Patients with severe aphasia, cognitive impairment, or consciousness disturbance who are unable to cooperate with the examination: These conditions can affect patients’ cooperation and the accuracy of the examination results. Therefore, to ensure the reliability of the research data and the safety of the patients, we excluded these patients. (2) Patients with incomplete or poor-quality fNIRS data: To ensure the accuracy and reliability of the research results, we excluded patients with incomplete or poor-quality fNIRS data. These situations include missing signal integrity, a low signal-to-noise ratio, data consistency issues, correctable artifacts, and data that do not meet the requirements for standardized processing. We assessed data quality through visual inspection, automated quality control metrics, and expert evaluation to ensure that only high-quality data were included in the analysis, thereby enhancing the credibility and effectiveness of the research results. On the basis of these inclusion and exclusion criteria, a total of 275 patients were enrolled in the study. Among these cases, 91 cases were classified as mild to moderate impairment (MI), and 184 cases were classified as severe impairment (SI). The patients were randomly allocated into a training group (*n* = 193, with 63 having MI and 130 with SI) and a validation group (*n* = 82, with 28 having MI and 54 with SI) based on a 7:3 ratio. Figure 1 illustrates the study’s flow diagram.

### 2.2 Data collection

The data collected for this study comprise two primary components. The initial component involves the retrieval and extraction of patients’ demographic characteristics from the electronic medical records system. These characteristics included age, sex, blood pressure at admission, duration poststroke, body mass index (BMI), presence of diabetes, hypertension, affected hemisphere, coronary heart disease, and Fugl-Meyer Assessment for Upper Extremity (FMA-UE) scores at admission. The second component pertains to the acquisition of 5-minute resting-state brain functional connectivity data via fNIRS. This includes the quantification of whole-brain functional connection edges (total edges of deoxyhemoglobin [HbD], total edges of oxyhemoglobin [HbO], total edges of total hemoglobin [HbT]) and the measurement of Short1 of edges (Short1 of edges for HbD/HbO/HbT), Short2 of edges (Short2 of edges for HbD/HbO/HbT), Long1 of edges (Long1 of edges for HbD/HbO/HbT), Long2 of edges (Long2 of edges for HbD/HbO/HbT), and the number of functional connection edges within 12 regions of interest (HbD/HbO/HbT in affected/unaffected primary motor cortex [PMC]/supplementary motor area [SMA]/dorsolateral prefrontal cortex [DLPFC]/temporal/frontopolar) and the number of connection edges between bilaterally symmetrical regions of interest (HbD/HbO/HbT in affected PMC/SMA/DLPFC/Temporal/Frontopolar-unaffected PMC/SMA/DLPFC/Temporal/Frontopolar), yielding a total of 69 fNIRS variables. Deoxyhemoglobin (HbD) and oxyhemoglobin (HbO) are key indicators of brain oxygenation and blood flow, respectively. Their measurements can provide insights into brain function and connectivity. Total hemoglobin (HbT) represents the total amount of hemoglobin in the blood, which can be used to assess overall cerebral blood volume.

Functional Near-Infrared Spectroscopy (fNIRS) data were acquired with the BS-2000 near-infrared spectroscopy system (Wuhan Zilian Hongkang Technology Co., Ltd., Wuhan, China). The instrument employs dual-wavelength laser diodes (690 nm and 830 nm) operating at a sampling rate of 10 Hz and is equipped with 32 sources and 32 detectors, forming 106 channels. Each channel consists of a source–detector pair separated by 3 cm. Optode placement followed the international 10–20 system, with source S2 positioned at Fpz. A 3-D digitizer (NirMap, Wuhan Zilian Hongkang Technology Co., Ltd.) was used to record the reference landmarks (Nz, Cz, AL, RL) and the spatial coordinates of all optodes. Channel locations were projected onto the cortical surface and assigned to the corresponding Brodmann areas using the NIRS-SPM approach (Liu et al., 2025).

### 2.3 Data analysis

The raw fNIRS data were preprocessed using the HOMER2 toolbox (version 2.8) integrated with MATLAB R2014b (MathWorks, Natick, MA, USA). The preprocessing steps included data conversion, segmentation, quality control, and signal processing. For detailed information on the preprocessing steps and parameter settings, including motion correction thresholds, please refer to the Supplementary Methods.

### 2.4 Model construction

The statistical analysis was conducted utilizing SPSS (IBM Corp, version 27.0) and R (version 4.2.1). This study undertook a comprehensive examination to identify predictors of upper limb motor dysfunction poststroke, employing fNIRS data. The normality of continuous variables was initially evaluated using the Kolmogorov-Smirnov test. The results indicated that none of the variables followed a normal distribution. Consequently, these variables were described using their median and interquartile range, and group comparisons were performed using the Mann-Whitney U test. Categorical variables are represented as frequencies (percentages) and were analyzed using chi-square tests, with statistical significance established at a p value of less than 0.05 (two-tailed).

The LASSO (Least Absolute Shrinkage and Selection Operator) regression model was used to identify variables correlated with poststroke upper limb motor dysfunction scores within the training dataset. LASSO regression is a statistical method that performs both variable selection and regularization to enhance the prediction accuracy and interpretability of the model. To determine the optimal penalty parameter (λ) for LASSO regression, we employed the method of K-fold cross-validation (commonly *K* = 10). In this process, the dataset was divided into K equal parts. K-1 parts were used for model training, and the remaining part was used for validating the model’s performance. This process is repeated K times, with a different part selected as the validation set each time, ensuring that every data point is used for validation once. Using this method, we assessed the model performance for different λ values and selected the λ value that minimizes the cross-validation error, thereby balancing the model’s bias and variance. After that, a logistic regression analysis was conducted, with poststroke upper limb motor dysfunction scores as the dependent variable and the variables selected by the LASSO regression as independent variables, to identify independent risk factors. To ascertain the robustness of the model, multicollinearity among the variables was evaluated through the application of Variance Inflation Factor (VIF) analysis.

A predictive nomogram was developed utilizing the independent risk factors identified through logistic regression analysis to estimate the likelihood of poststroke upper limb motor dysfunction. This nomogram functions as a visual tool for clinicians to assess the risk of motor dysfunction on the basis of individual patient characteristics. The calibration of the model was evaluated through calibration curve analysis to verify that the predicted probabilities aligned with the observed outcomes. Additionally, the clinical utility of the model was assessed using Decision Curve Analysis (DCA), which quantifies the net benefit by balancing potential harms against benefits. DCA is a method that evaluates the net benefit of a predictive model by comparing the benefits of true positive predictions against the harms of false positive predictions. The sensitivity, specificity, and negative predictive value are used to evaluate the performance of the model, whereas the Hosmer-Lemeshow test is used to detect the calibration of the model.

All analyses were conducted utilizing R version 4.2.1, which employs a range of specialized packages for distinct analytical tasks. The “glmnet” package was used for LASSO regression to identify predictive variables, whereas the “car” package facilitated VIF analysis to assess multicollinearity. The “rms” package was employed in the construction of the predictive nomogram, and “pROC” was used to calculate the C-index for evaluating model discrimination. Additionally, “PredictABEL” was utilized to assess improvements in predictive performance, including Integrated Discrimination Improvement (IDI) and Net Reclassification Improvement (NRI). The “rmda” package was applied for DCA to evaluate the clinical utility of the developed nomogram.

## 3 Results

### 3.1 Baseline characteristics

After applying rigorous inclusion and exclusion criteria, 275 patients were deemed eligible for study inclusion. The patients’ baseline characteristics are detailed in Table 1. The participants were subsequently divided into “(*n=193*, see Supplementary Table 2) and a validation group (*n=82*, see Supplementary Table 1).” The comparative baseline characteristics of these two groups are presented in Table 2. Statistical analyses revealed no significant differences in demographic variables, including sex, age, disease progression, medical history, or other relevant factors, between the training and validation groups (*P* > 0.05). This result highlights the methodological rigor of the randomization process, ensuring that the allocation of patients across groups was both scientifically valid and equitable.

**TABLE 1:** Baseline characteristics of subjects.

Variables	Total (*N* = 275)	MI (*N* = 84)	SI (*N* = 191)	χ ^2^/Z	*P*
Sex, *n* (%)				0.256	0.613^a^
Male	134 (48.7)	39 (46.43)	95 (49.74)		
Female	141 (51.27)	45 (53.57)	96 (50.26)		
High blood pressure, *n* (%)				0.319	0.572^a^
Yes	177(64.36)	52(61.90)	125(65.45)		
No	98(35.64)	32(38.10)	66(34.55)		
Heart disease, *n* (%)				0.732	0.392^a^
Yes	102(37.09)	28(33.33)	74 (38.74)		
No	173(62.91)	56 (66.67)	117 (61.26)		
Diabetes, *n* (%)				0.173	0.678^a^
Yes	159 (57.82)	47 (55.95)	112 (58.64)		
No	116 (42.18)	37 (44.05)	79 (41.36)		
History of stroke, *n* (%)				1.277	0.259^a^
Yes	43 (15.64)	10 (11.90)	33 (17.28)		
No	232 (84.36)	74 (88.10)	158 (82.72)		
Diseased hemisphere, *n* (%)				0.035	0.852^a^
Left	191 (69.45)	59 (70.24)	132 (69.11)		
Right	84 (30.55)	25 (29.76)	59 (30.89)		
Age (years)	66 (59,72)	64 (57,72)	66 (60,72)	−1.639	0.101^b^
BMI (kg/m2)	24.38 (23.36,25.43)	24.67 (23.23,25.69)	24.36 (23.36,25.39)	−0.723	0.470^b^
CD (days)	52 (30,86)	46.5 (22.25,80.75)	56 (31,86)	−1.469	0.142^b^
Heart rate (bpm)	76 (70,81)	77 (69.25,82)	75 (71,80)	−0.837	0.403^b^
SP (mmHg)	123 (115,131)	126 (118,133)	122 (113,131)	−1.308	0.191^b^
DP (mmHg)	77 (68,84)	76 (67,83)	77 (69,84)	−1.062	0.288^b^
ADL	25 (0,50)	55 (50,70)	5 (0,25)	−13.313	0.000^b^

CD, courses of disease; SP, systolic pressure; DP, diastolic pressure. *^a^*Using the χ^2^ test, *^b^*Using the Mann-Whitney U test.

**TABLE 2:** The baseline characteristics of the patients in the training and validation cohort.

Variables	Total (*N* = 275)	Training (*N* = 192)	Validation (*N* = 83)	χ ^2^/Z	*P*
Diagnosis, *n* (%)				1.082	0.298^a^
MI	84 (30.55)	55 (28.65)	29 (34.9)		
SI	191 (69.45)	137 (71.35)	54 (65.1)		
Sex, *n* (%)				0.412	0.521^a^
Male	134 (48.7)	96 (50)	38 (45.78)		
Female	141 (51.27)	96 (50)	45 (54.22)		
High blood pressure, *n* (%)				0.025	0.874 ^a^
Yes	177(64.36)	123 (64.06)	54 (65.06)		
No	98(35.64)	69 (35.94)	29 (34.94)		
Heart disease, *n* (%)				0.236	0.627^a^
Yes	102(37.09)	73 (38.02)	29 (34.94)		
No	173(62.91)	119 (61.98)	54 (65.06)		
Diabetes, *n* (%)				0.632	0.427^a^
Yes	159 (57.82)	114 (59.38)	45 (54.22)		
No	116 (42.18)	78 (40.63)	38 (45.78)		
History of stroke, *n* (%)				0.512	0.474^a^
Yes	43 (15.64)	32 (16.67)	11 (13.25)		
No	232 (84.36)	160 (83.33)	72 (86.75)		
Diseased hemisphere, *n* (%)				0.034	0.854^a^
Left	191 (69.45)	134 (69.79)	57 (68.67)		
Right	84 (30.55)	58 (30.21)	26 (31.33)		
Age (years)	66 (59,72)	66 (59,72.75)	64 (59,72)	−0.966	0.334^b^
BMI (kg/m2)	24.38 (23.36,25.43)	24.27 (23.45,25.43)	24.22 (23.25,25.38)	−1.444	0.149^b^
CD (days)	52 (30,86)	55 (30,86)	46 (30,80)	−1.034	0.301^b^
Heart rate (bpm)	76 (70,81)	76 (70,82)	76 (70,79)	−0.533	0.594^b^
SP (mmHg)	123 (115,131)	124 (115,132.75)	123 (115,131)	−0.689	0.491^b^
DP (mmHg)	77 (68,84)	77 (69,84.75)	75 (66,82)	−1.894	0.058^b^
ADL	25 (0,50)	25 (0,50)	25 (0,55)	−0.676	0.499^b^

CD, courses of disease; SP, systolic pressure; DP, diastolic pressure. *^a^*Using the χ^2^ test, *^b^*Using the Mann-Whitney U test.

### 3.2 Nomogram construction

**FIGURE 2:**
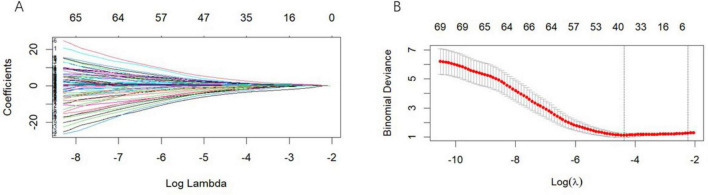
LASSO regression analysis with 10-fold cross-validation for predicting motor functional impairment post-stroke. **(A)** Use LASSO regression to make a map ofrisk factor coefficients. **(B)** Preliminary screening of clinical risk factors using LASSO regression.

Figures 2A, B display the outcomes of the LASSO regression feature selection process. Figure 2A provides a visualization of the LASSO regression coefficient plot for all risk factors, with each curve representing a unique risk factor. The vertical axis indicates the regression coefficient of the predictor, whereas the horizontal axis represents the logarithm of λ. In contrast, Figure 2B illustrates the binomial deviance, where the lowest point on the curve signifies the optimal parameter λ. In this study, the application of LASSO regression resulted in the identification of five variables integral to the evaluation of poststroke motor dysfunction. These variables include Changes in HbD in the affected temporal region, Total HbT levels, HbT affecting the DLPFC, HbT levels in the unaffected frontopolar region, and HbT Short1 of the edges.

**FIGURE 3:**
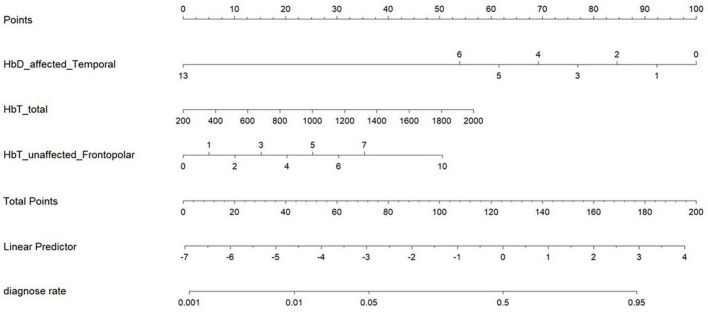
The nomogram. This nomogram is based on three independent risk factors: HbD levels in the affected temporal region, total HbT levels, and HbT levels in the unaffected frontopolar region. To use the nomogram, follow these steps: (1) Locate the value of each risk factor on the corresponding axis (for example, HbD_affected_Temporal = 13, HbT_total = 500, HbT_unaffected_Frontopolar = 2). (2) Draw a vertical line upward from each value to the “Points” axis to determine the score for each factor. (3) Add up the scores for all three factors (for example, HbD score = 0, HbT_total score = 10, HbT_frontopolar score = 10; total score = 20). (4) Draw a vertical line upward from the total score to the “diagnose rate” axis to obtain the predicted probability of upper limb motor dysfunction (for example, the predicted probability corresponding to a total score of 20 is 0.004). Example: For a patient with HbD_affected_Temporal = 13, HbT_total = 500, and HbT_unaffected_Frontopolar = 2, the nomogram predicts a probability of upper limb motor dysfunction of 0.4%.

A logistic regression analysis was performed to determine the weights and *p*-values of each predictive variable identified through LASSO regression, using a significance threshold of *p* < 0.05. As presented in Table 3, the variables HbT affected DLPFC and HbT Short1 of edges did not meet the significance criteria (*P* > 0.05) and were therefore excluded from further consideration. The exclusion of these variables was based on their lack of statistical significance, which suggests that they may not contribute substantially to the prediction of upper limb motor dysfunction scores following ischemic stroke. However, it is important to note that the choice of *p* < 0.05 is a common convention in medical research. Consequently, three independent risk factors associated with upper limb motor dysfunction scores following ischemic stroke were identified: Changes in HbD in the affected temporal region, Total HbT levels, and HbT levels in the unaffected frontopolar region, all of which satisfied the condition of p < 0.05 (refer to Table 4). On the basis of these three risk factors, a nomogram was developed for predictive purposes, as detailed in Figure 3.

**TABLE 3:** Original model coefficients.

Variables	β	SE	OR	CI	*P*-value
Intercept	−2.604	0.597	0.073	0.004, 0.135	<0.001
HbD affected temporal	−0.444	0.117	0.641	0.508, 0.806	<0.001
HbT total	0.001	0.001	1.001	0.995, 1.007	0.037
HbT affected DLPFC	0.062	0.042	1.063	0.980, 1.153	0.143
HbT unaffected frontopolar	0.231	0.103	1.261	1.070, 1.484	0.025
HbT Short1 of edges	0.011	0.007	1.011	0.997, 1.026	0.131

**TABLE 4:** Final model coefficients.

Variables	β	SE	OR	CI	*P*-value
Intercept	−1.747	0.436	0.175	(0.032,0.937)	<0.001
HbD affected temporal	−0.435	0.115	0.648	(0.370,1.136)	<0.001
HbT total	0.002	0.001	1.002	(0.996,1.007)	<0.001
HbT unaffected frontopolar	0.285	0.099	1.331	(0.992,1.793)	<0.001

### 3.3 Model validation

**FIGURE 4:**
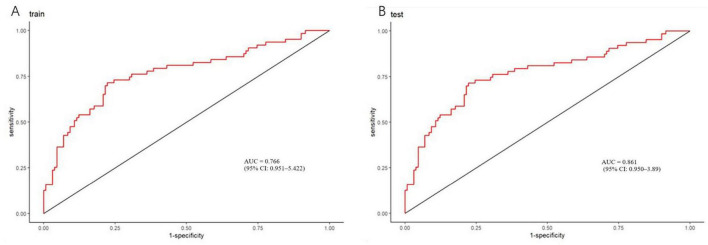
Receiver operating characteristic (ROC) curves for nomogram. **(A)** Training ROC. This graph illustrates the ROC curve of the model on the training dataset. The area under the curve (AUC) is 0.766 (95% confidence interval: 0.951–5.422), indicating a moderately high discriminative power of the model in distinguishing between the presence and absence of poststroke upper limb motor dysfunction. This suggests that the model can identify patients with motor dysfunction with relatively good accuracy. The *x*-axis represents the false positive rate (1 - specificity), and the *y*-axis represents the true positive rate (sensitivity). The diagonal line indicates a model with no discriminative ability (AUC = 0.5). The closer the ROC curve is to the top-left corner, the better the model’s performance. **(B)** Validation ROC. This graph displays the ROC curve of the model on the test dataset. The AUC is 0.861 (95% confidence interval: 0.950–3.89), demonstrating that the model maintains high discriminative power on new, unseen data. This result indicates that the model has good generalization ability and can effectively identify poststroke upper limb motor dysfunction in practical applications.

**FIGURE 5:**
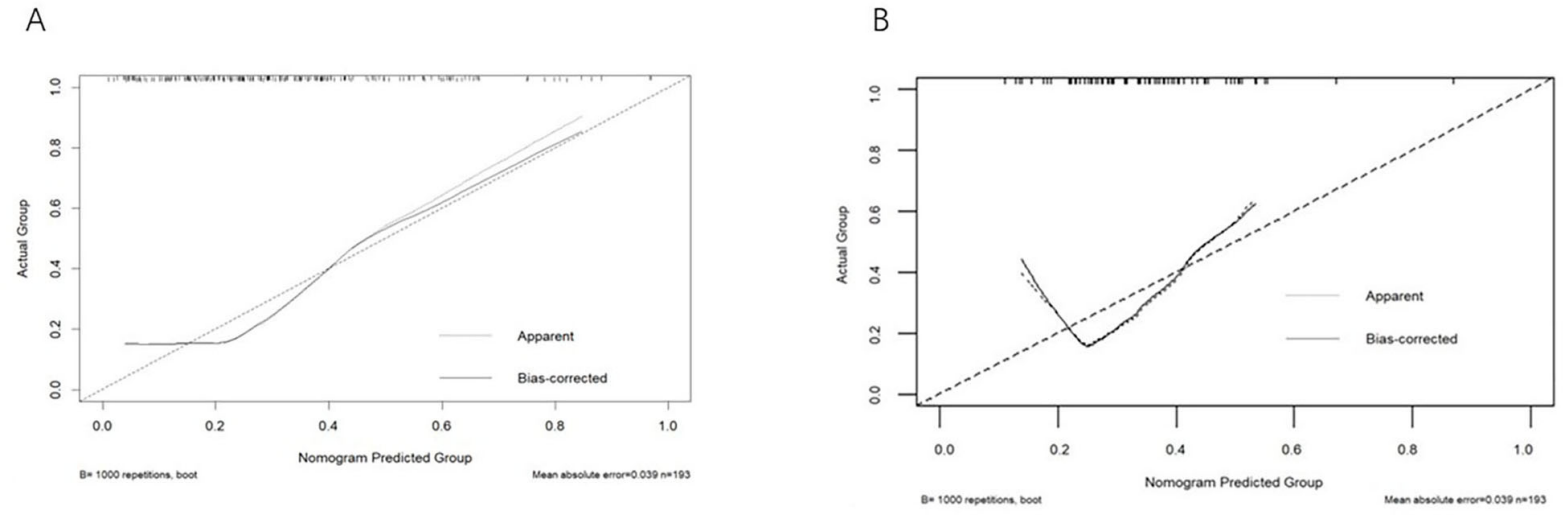
Calibration curves for the training cohort and the validation cohort. **(A)** Training cohort. The calibration curve for the training cohort shows the agreement between the predicted probabilities of upper limb motor dysfunction and the observed outcomes. The *x*-axis represents the predicted probability, and the *y*-axis represents the actual observed probability. The dashed diagonal line represents perfect calibration, where predicted probabilities perfectly match observed outcomes. The solid line represents the model’s calibration, with closer alignment to the diagonal indicating better calibration. **(B)** Validation cohort. The calibration curve for the validation cohort similarly compares predicted and observed probabilities. The *x*-axis and *y*-axis are labeled as in **(A)**. The close alignment of the solid line to the diagonal indicates that the model is well-calibrated and generalizes well to new data.

**FIGURE 6:**
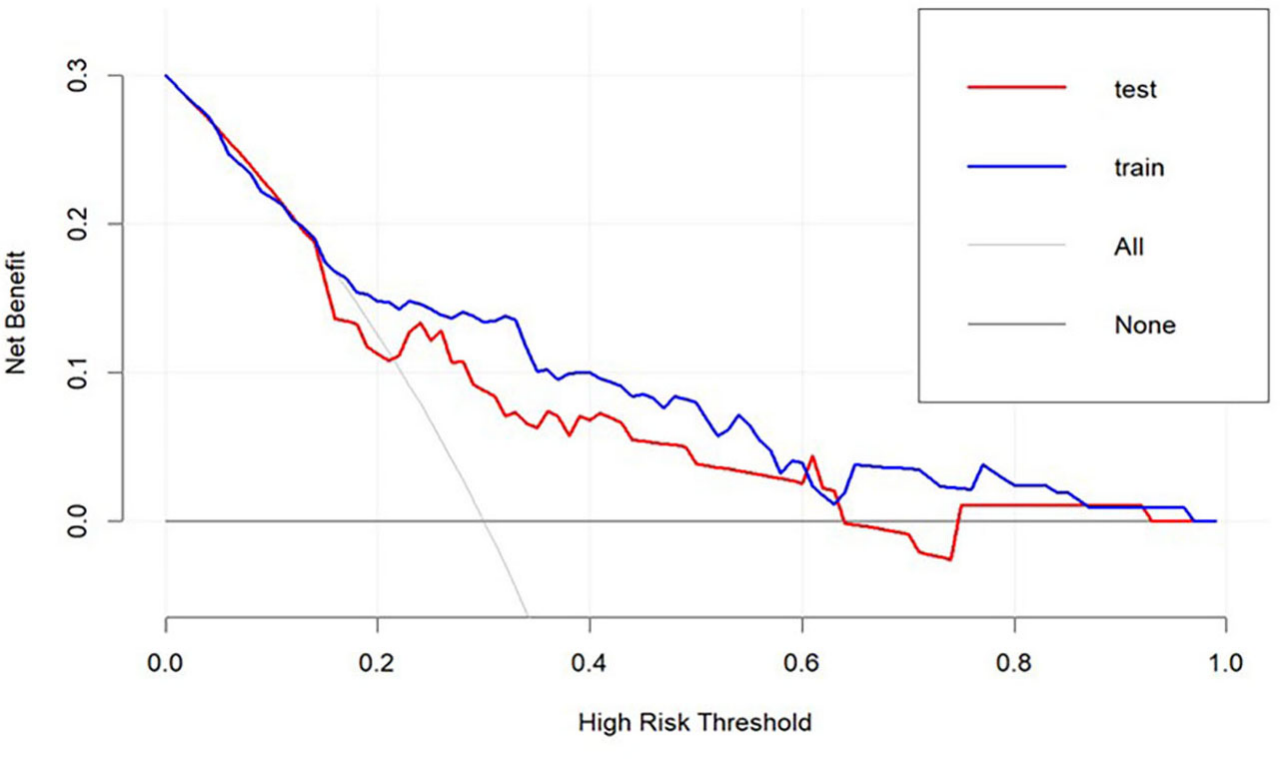
Decision-curve analysis.

To assess the discriminative ability of our model, we computed the area under the receiver operating characteristic (ROC) curve (AUC). As depicted in Figure 4A, the AUC for the training dataset was 0.766 (95% CI: 0.951–5.422). Figure 4B shows that the AUC for the testing dataset was 0.861 (95% CI: 0.950–3.89). For the training set, the sensitivity was 0.429, the specificity was 0.915, the negative predictive value was 0.768, and the p value for the Hosmer-Lemeshow test was 0.125. For the validation set, the sensitivity was 0.357, the specificity was 0.889, the negative predictive value was 0.727, and the Hosmer-Lemeshow test *p*-value was 0.211. Overall, the model exhibited satisfactory performance across both the training and validation datasets. It demonstrated high specificity, reflecting a strong ability to accurately identify non-target classes, and maintained a reasonable negative predictive value. The p values from the Hosmer-Lemeshow test exceeded 0.05 for both datasets, indicating a good fit of the model. Refer to Figure 5 for further details. Figure 6 illustrates the results of the DCA for the model. These results of the DCA demonstrated a substantial clinical net benefit in both the validation and development groups. This is observed across threshold probability ranges of 0.05–0.95 for the training dataset and 0.05–0.95 for the testing dataset (refer to Figure 6).

### 3.4 Correlation with ADL scores

We conducted a correlation analysis between the identified model variables and the activities of daily living (ADL) scores of the patients (refer to Table 5 Correlation Results between Model Variables and ADL). The results showed no significant correlation between the model variables and ADL scores (*p* > 0.05 for all variables). Specifically, the HbD levels in the affected temporal region (rs = −0.061, *p* = 0.315), the total HbT levels (rs = −0.022, *p* = 0.721), and the HbT levels in the unaffected frontopolar region (rs = 0.077, *p* = 0.202) did not exhibit significant associations with ADL scores. This suggests that while these variables are predictive of upper limb motor dysfunction severity, they may not directly influence the patients’ ability to perform daily living activities.

**TABLE 5:** Correlation results between model variables and ADL.

	ADL (rs, p)
HbD affected temporal	−0.061, 0.315
HbT total	−0.022, 0.721
HbT unaffected frontopolar	0.077, 0.202

## 4 Discussions

In this study, three independent risk factors were identified as being associated with the severity of upper limb motor dysfunction after ischemic stroke: (1) Changes in HbD in the affected temporal region, (2) Total HbT levels, and (3) HbT levels in the unaffected frontopolar region. Our model demonstrated good discriminative power in both the training and validation datasets, with AUC values reaching 0.766 and 0.861, respectively. This result underscores the efficacy and reliability of the model in clinical settings. Moreover, the nomogram we developed provides clinicians with an intuitive tool for objectively assessing the functional status of stroke patients, which can aid in devising personalized treatment plans and offering patients more precise diagnostic information.

However, it is important to note that our analysis of the correlation between model variables and ADL scores showed no significant correlation (with all variables having *p* > 0.05). This indicates that although the identified risk factors can be used to predict the severity of upper limb motor dysfunction, they may not be directly related to patients’ functional outcomes in daily activities. This finding highlights the complexity of post-stroke rehabilitation, where many other factors, in addition to hemodynamic changes in specific brain regions, may influence ADL performance. This phenomenon may be influenced by several factors: Firstly, the complexity of neuroplasticity itself cannot be ignored. After a stroke, the brain undergoes a highly complex process of neuroplasticity (Xi et al., 2023, Chen et al., 2023, Cramer et al., 2023), with dynamic changes occurring in multiple brain regions and neural networks (Yao et al., 2020, Liu et al., 2025). The model variables included in this study mainly reflect the correlation signals between brain regions related to function. These signal changes may not correspond completely with the improvement in patients’ motor function or daily living abilities as assessed by clinical scales (Arun et al., 2020, Huo et al., 2023). Secondly, there are significant individual differences among stroke patients. Factors such as lesion location, lesion size, and neural recovery potential vary from person to person, which may lead to inconsistencies in the relationship between brain functional connectivity and clinical scales (Liu et al., 2025).

Lastly, the limitations of measurement methods may also be a reason. Brain functional connectivity is measured using fNIRS technology to assess hemodynamic changes in the cerebral cortex, while the clinical scale (ADL) evaluates patients’ motor function and daily living abilities through behavioral assessment. The differences in sensitivity and specificity between these two measurement methods may result in a lack of significant correlation between them (Liu et al., 2025, Yang and Wang, 2025).

The HbD and HbT levels identified in this study as important predictive indicators hold significant neurophysiological importance. The changes in HbD levels in the affected temporal region may reflect the metabolic demands of local brain areas associated with motor function. Neural activity and energy metabolism in the brain are closely related to changes in HbD. For example, Highton’s article (Highton et al., 2012) indicates that deoxyhemoglobin serves as a biomarker of cerebral autoregulation. The total HbT level, on the other hand, reflects overall cerebral blood volume, which is crucial for maintaining normal neurological function. Additionally, the HbT level in the unaffected frontal pole region may be associated with compensatory mechanisms in the brain following a stroke. Research has shown that the frontal pole plays a role in motor coordination and recovery.

Regarding the calibration curves and decision curve analysis (DCA), our results indicate that the model is well-calibrated, with predicted probabilities closely aligning with observed outcomes. The DCA further supports the model’s clinical utility by demonstrating a favorable net benefit across a range of threshold probabilities (0.05–0.95). This suggests that the model could be clinically useful in guiding treatment decisions, particularly in identifying patients who may benefit from targeted rehabilitation programs. However, we recognize that the DCA results are based on a single-center dataset, and future studies should validate these findings in multi-center settings to ensure broader applicability.

To ensure the robustness of the model, multicollinearity among the variables was evaluated using Variance Inflation Factor (VIF) analysis. VIF analysis is a statistical method used to detect the presence of multicollinearity in a set of multiple regression variables. A VIF value greater than 10 is generally considered to indicate high multicollinearity, which can distort the regression coefficients and make the model less reliable. In our study, all VIF values were less than 10, indicating that multicollinearity was not a significant issue in our model. This ensures that the predictors included in the model are independent and contribute uniquely to the prediction of upper limb motor dysfunction scores.

Unlike Ye (Ye et al., 2024), who used a linear regression model combining sEMG and fNIRS signals, we employed LASSO regression and logistic regression analysis to identify biomarkers with higher discriminative power. Compared to Zhou (Zhou et al., 2024), who used machine learning models to predict recovery, we used statistical methods to identify risk factors. These differences highlight the innovation in our assessment methods and model construction, offering new perspectives and tools for stroke rehabilitation assessment.

To address potential concerns regarding the adequacy of our sample size, we conducted power analyses based on the AUC values obtained from both the training and validation datasets. The results indicated that our sample sizes were adequate to support the model’s discriminative ability, with power nearing 100% in both datasets. Specifically, in the training dataset, which comprised 130 positives and 63 negatives with an AUC of 0.766, the power was nearly 100%. Similarly, in the validation dataset, consisting of 54 positives and 28 negatives with an AUC of 0.861, the power was also close to 100%. These findings confirm that our chosen sample size was appropriate for detecting the expected effect sizes, ensuring robust and statistically significant results for both model development and validation.

Our findings reveal the key roles of the temporal lobe and the frontal pole in stroke recovery. Regarding the role of the temporal lobe in the recovery of motor function in stroke patients, studies by Kenzie (Kenzie et al., 2019) and Liu (Liu et al., 2021) have shown that the temporal lobe plays a significant role in the recovery of proprioception, whereas the frontal pole is crucial for improving motor coordination, especially in walking ability (Berger et al., 2019, Chatterjee et al., 2019). This is closely related to the recovery of motor function in stroke patients. This finding is also consistent with the results of our study. Our findings further support that changes in functional connectivity are not limited to between brain regions but also include within brain regions, reflecting that recovery after a stroke is a multifaceted process.

The current model has an AUC of 0.766 in the training set and 0.861 in the validation set, with sensitivity at 0.429 in the training set and 0.357 in the validation set, which indeed indicates potential for optimization. Considering the focus and current progress of this study, we did not attempt to use other machine learning algorithms (such as random forest and support vector machines) to optimize the model performance this time. However, we have acknowledged this as a limitation of our study in the discussion section and plan to explore these algorithms in future research to see if they can improve the model’s predictive accuracy and sensitivity. Meanwhile, to enhance the robustness of the model, we have used stricter cross-validation techniques to improve feature selection. In determining the optimal penalty parameter (λ) for LASSO regression, we employed a more rigorous repeated sampling 10-fold cross-validation method, which was repeated multiple times to ensure the stability of the selected λ value.

This study employed LASSO regression for variable selection because of its ability to reduce the risk of model overfitting and enhance the accuracy of predictions. However, this method may be sensitive to outliers and has a certain dependence on parameter settings. On the other hand, logistic regression analysis, on the other hand, provides us with a robust framework to assess the relationships between risk factors and ULMD-IS.

Although this study utilized MATLAB R2014b for data processing, the algorithms and methods employed remain universally applicable and reliable in subsequent versions. Furthermore, we will explore the use of updated software versions for data processing in future studies to verify the consistency and stability of the results, as well as to enhance the timeliness of our research.

Our model not only performs well statistically but also has practical clinical application value. By collecting resting-state fNIRS data from admitted patients and subsequently identifying the three key data points—HbD levels in the affected temporal region, HbT levels in the total region, and HbT levels in the unaffected frontopolar region—clinicians can more accurately and objectively differentiate stroke patients with upper limb motor dysfunction at different levels, formulate personalized diagnosis and rehabilitation plans, and potentially improve patients’ long-term outcomes.

Although our study provides valuable insights, it is not without limitations. For example, although the AUC value is acceptable, it is not particularly high, indicating that there is room for further optimization. One potential reason for this situation could be the insufficient sample size and reliance on data from a single source, which may undermine the model’s predictive accuracy and reliability. To increase the model’s universality and robustness, future research should expand the sample size and incorporate data from multiple research centers. Additionally, the limitations of the current study include its retrospective design, which may restrict the broad applicability of the results. The small sample size may affect the model’s stability and predictive power, and potential biases may also interfere with the interpretation of the results. To address issues such as sample size and single - source data, we propose the following plans for future research. First, for multi - center validation, we will collaborate with multiple research centers to collect a larger and more diverse dataset. This will not only increase the sample size but also enhance the representativeness of the data, thereby improving the model’s universality and robustness. Second, for model optimization, we will explore different machine - learning algorithms and techniques. For example, we may attempt neural network - based algorithms to see if we can further improve the model’s predictive accuracy. Additionally, we will conduct sensitivity analyses to better understand the impact of different variables on the model’s performance and carry out targeted optimizations. Regarding the clinical value, we plan to integrate the model into the clinical workflow. We will cooperate with developers of electronic medical record systems to create an interface that can automatically import relevant fNIRS data from patients’ records. This will enable clinicians to quickly obtain the model’s prediction results and incorporate them into their daily decision - making processes. For instance, when a stroke patient is admitted, the system can immediately calculate and assess the severity of upper limb motor dysfunction based on the collected fNIRS data, providing clinicians with timely and useful information for formulating treatment plans.

The results of this study may have a positive impact on patient management by providing more accurate diagnostic information, which can help patients and doctors make more effective treatment decisions. However, any decision based on predictive models should take into account individual differences and be used under the guidance of clinical judgment. Our findings provide novel insights into the assessment and management of upper limb motor dysfunction subsequent to ischemic stroke, thereby serving as valuable references for future research and clinical practice.

## 5 Conclusion

In this investigation, the application of LASSO regression and logistic regression analyses enabled the identification of three independent risk factors correlated with the severity of upper limb motor dysfunction subsequent to ischemic stroke: HbD levels in the affected temporal region, HbT levels in the total region, and HbT levels in the unaffected frontopolar region. A nomogram model was subsequently developed based on the basis of these identified factors. Although the model exhibited strong discriminative ability, with high specificity and an acceptable negative predictive value, its sensitivity was low. The model’s reliability and clinical net benefit were validated using the Hosmer-Lemeshow test and decision curve analysis, confirming its effectiveness in predicting upper limb motor function scores in stroke patients. In settings where high sensitivity is not critical, this model may assist clinicians in risk assessment and treatment planning and could, if used judiciously, contribute to modest improvements in patient outcomes and some reduction in long-term disability-related costs. It also lays the groundwork for future research, suggesting the need for model validation across different populations and exploring its integration into electronic medical records to enhance the clinical workflow and the model’s utility in everyday practice. Overall, our study not only advances the understanding of poststroke motor dysfunction but also provides a practical tool that could influence patient management and guide future research directions in the field of stroke rehabilitation.

## Data Availability

The original contributions presented in this study are included in this article/Supplementary material, further inquiries can be directed to the corresponding author.
