# Supplementary material for: Predictive nomogram for post-stroke motor dysfunction using fNIRS
Source: Front Hum Neurosci. 2025 Dec 4;19:1526455. doi: 10.3389/fnhum.2025.1526455 (PMC12711766; doi:10.3389/fnhum.2025.1526455)
Supplement: Supplementary file 3 [file Table_3.docx]

Data Preprocessing with HOMER2:

Data Conversion: The initial data format was transformed from the Comma Separated Values (CSV) format to the nirs format to facilitate subsequent analytical procedures.

Data Segmentation: A specific temporal window, spanning from the 25th second to the 300th second, was extracted to concentrate on the pertinent segment for analysis.

Quality Control: Stringent quality control protocols were established, setting a threshold at 25% to ensure exclusive inclusion of high-fidelity data segments.

Signal Processing:

The initial near-infrared light intensity measurements were transformed into optical density (OD) signals to standardize the results for subsequent analysis.

Motion Artifact Detection: Motion artifact detection was conducted across all channels utilizing the built-in functions of the HOMER2 toolbox, with the following parameter settings: tMotion=0.5 seconds, tMask=3.0 seconds, STDEVthresh=20.0, and AMPthresh=5.0.

The hmrMotionCorrectSpline method was employed to detect and rectify genuine motion artifacts via spline interpolation.

A bandpass filter ranging from 0.01 to 0.1 Hz was applied to the dataset to effectively remove the majority of systemic hemodynamic components, including those associated with the cardiac cycle (approximately 1 Hz) and respiration (approximately 0.2–0.3 Hz).

The filtered optical density data were then transformed into concentrations of HbO, HbD, and HbT through the application of the modified Beer-Lambert Law, thereby yielding quantitative measurements of blood.

Motion Correction Thresholds:

The motion correction threshold was set at 0.5 mm, based on previous studies and the recommendation of the HOMER2 toolbox. Any segments with motion artifacts exceeding this threshold were corrected using spline interpolation to minimize the impact of motion on the data quality.
